# Role of extrinsic mechanical force in the development of the RA-I tactile mechanoreceptor

**DOI:** 10.1038/s41598-018-29390-x

**Published:** 2018-07-23

**Authors:** Trung Quang Pham, Takumi Kawaue, Takayuki Hoshi, Yoshihiro Tanaka, Takaki Miyata, Akihito Sano

**Affiliations:** 1 Robotics Lab, Department of Electrical and Mechanical Engineering, Graduate School of Engineering, Nagoya Institute of Technology, Nagoya, 466-8555 Japan; 2 Pixie Dust Technologies, Inc., Tokyo, 101-0041 Japan; 3 Department of Anatomy and Cell Biology, Nagoya University, Nagoya, 466-8550 Japan

## Abstract

Rapidly adapting type I (RA-I) mechanoreceptors play an important role in sensing the low-frequency vibration aspects of touch. The structure of the RA-I mechanoreceptor is extremely complex regardless of its small size, limiting our understanding of its mechanotransduction. As a result of the emergence of bioengineering, we previously proposed an *in vitro* bioengineering approach for RA-I receptors to overcome this limitation. Currently, the *in vitro* bioengineering approach for the RA-I receptor is not realizable given the lack of knowledge of its morphogenesis. This paper demonstrates our first attempt to interpret the cellular morphogenesis of the RA-I receptor. We found indications of extrinsic mechanical force nearby the RA-I receptor in the developing fingertip. Using a mechanical compression device, the axon of dorsal root ganglion (DRG) neurons buckled *in vitro* into a profile that resembled the morphology of the RA-I receptor. This work encourages further implementation of this bioengineering approach in tactile receptor-related research.

## Introduction

Rapidly adapting type I (RA-I) mechanoreceptors, which are also called Meissner corpuscles (end organs), are one type of mechanoreceptor found underneath mammal skin. These receptors are located at the middle region between the epidermis and the dermis (dermal papillae). Although they play an important role in sensing the low-frequency vibration aspects of touch, their mechanotransduction is not completely understood. Most of the research concerning mechanotransduction of RA-I receptors has been conducted while including mechanical properties of the surrounding environment (skin), which are highly varied between species and individuals. This limitation still exists because the isolation of RA-I receptors is extremely difficult. Regardless of its small size, the structure of the RA-I receptor is the most complex among mechanoreceptors, consisting of spiral-like axons, lamellar cells (Schwann cells), and a collagen capsule (for anatomical details, please refer to^1,2^).

To overcome the existing limitation, we previously proposed a bioengineering approach by which RA-I receptor-like morphology could be represented *in vitro*^3^. Among the components of RA-I receptors, the configuration of the axon terminal is the most complex (spiral shape) and interesting to most researchers. How and why the receptor forms this complex configuration is not well understood. Histological observations reveal that axons of RA-I receptors begin at the somata, which lies in the dorsal root ganglion (DRG), and travel a long distance towards the fingertip. Despite the various periods of development between species, the first axon terminal is typically straight when it is innervated in the dermal papillae. After reaching the apex of the dermal papillae, this axon terminal continues to deform, whereas the development of its surrounding components (such as lamellar cells and collagen capsule) starts later^4,5^. The other axon terminals penetrate the receptor laterally and appear to follow the course of the first penetrated axon terminal. Inevitably, the deformation of the first penetrated axon terminal is essential for the development of RA-I receptors. Which factor (chemical or physical) causes the deformation of the first penetrated axon terminal is unclear.

In recent years, researchers have identified various factors that may alter the morphology of axon terminals *in vitro*. In the chemical category, chemical gradients and laser-induced photolysis have been used to control the concentration of attractive proteins nearby growth cones^6,7^. In the physical category, topographical^8^, electrical^9^, optical^10–13^, and microfluidic^14^ cues, as well as a hybrid approach, such as optofluidic flow^15,16^, have been employed for the purpose of axonal guidance. In recent studies, microfluidic flow has been applied directly to provide pressure on growth cones^14^; alternatively, a focused laser beam has been used to trap and rotate birefringent beads^15,16^, exerting shear force on growth cones. Both approaches have resulted in axonal growth control at a fine spatial resolution. These works demonstrate the promising ability to create the complex structure of an axon terminal exclusively using physical cues. Considering the morphology of axon terminals in RA-I receptors, a role for physical cues (i.e., mechanical force) in the deformation of the first penetrated axon terminal is reasonable to suspect.

In this study, we conducted a series of experiments to investigate the effect of mechanical forces on the deformation of axon terminals with regard to the development of the RA-I receptor. We determined the relationship between the compression of collagen fibres and the deformation of axonal terminals using two-photon microscopy on stained serial sections. These findings indicate the existence of extrinsic mechanical forces at the developing RA-I position. A stretchable chamber was used to provide extrinsic mechanical force to the axon terminals innervating a dermal papillary structure (made of silicone). We observed that the axon terminals deformed in the same manner as the developing RA-I receptors *in vivo*. Our findings help explain a portion of the morphogenesis of the RA-I receptors. Ultimately, the bioengineering approach described in this paper may provide a novel ability to examine the developmental and functional aspects of RA-I *in vitro*.

## Results

### Compression of collagen fibres at the base of dermal papillae during RA-I receptor development

**Figure 1:**
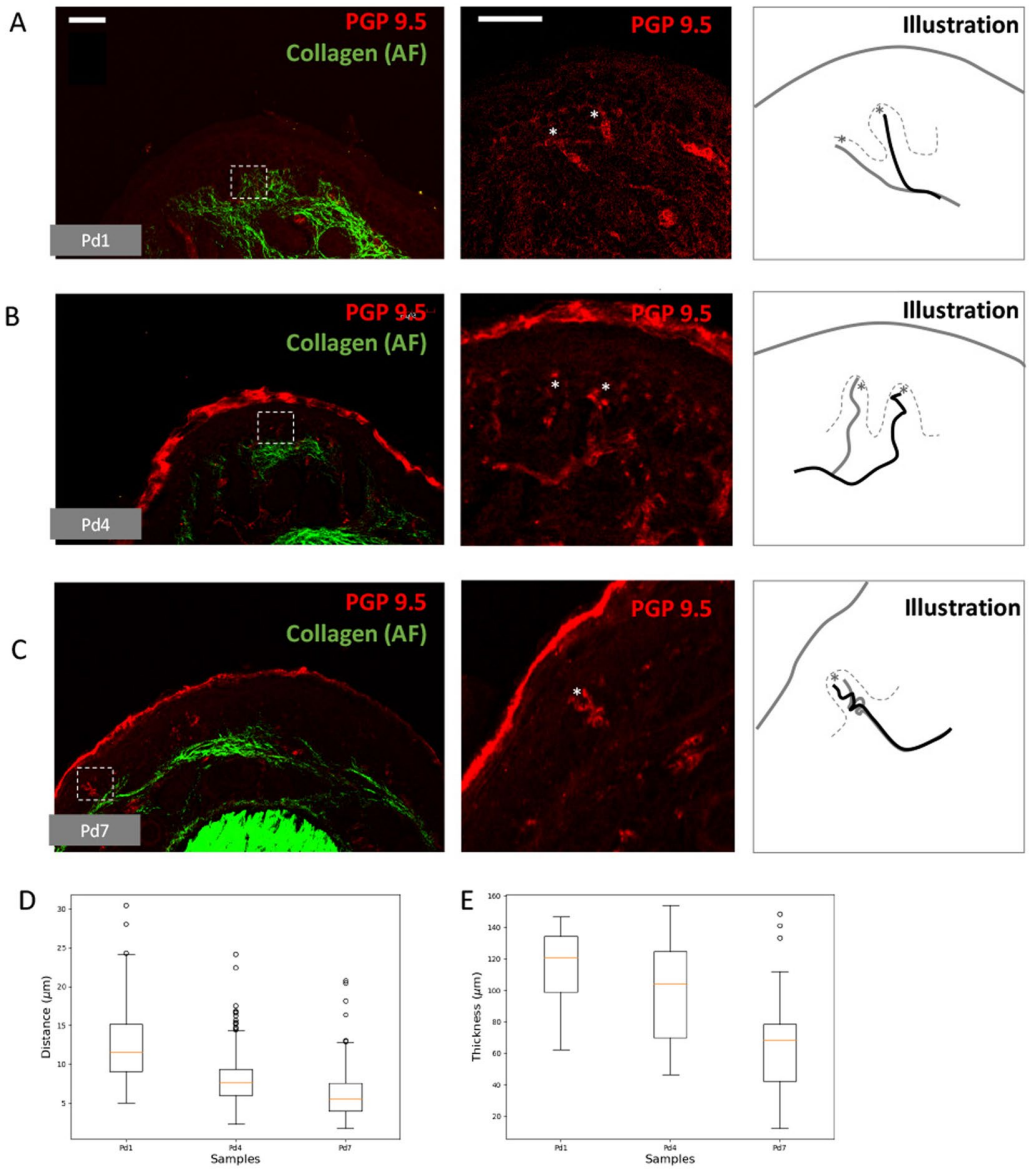
Immunostaining of collagen fibres (autofluorescent-green colour) and axon terminals (PGP 9.5-red colour). White bar = 50 *μ*m. (**A**–**C**) Sequential observations from Pd1 to Pd7 reveals deformation of innervated axon terminals. The dashed square indicates the region of interest, as shown in the middle image. The asterisks indicate the tips of innervating axon terminals. For better visualization, three illustrations were prepared with the dashed lines representing the dermal papillae ridges between epidermis and dermis. (**D**) Box plots demonstrate the change in distribution of distance between collagen fibres by day (Pd1, Pd4, and Pd7). The yellow lines indicate the medians of the distribution. (**E**) Box plots depict the change in distribution of dermal thickness by day (Pd1, Pd4, and Pd7).

In search for evidence of an extrinsic mechanical force on nearby RA-I receptors in the developing mouse fingertip, we used an immunostaining method on serial sections with the plane perpendicular to the longitudinal axis of finger (postnatal-day (Pd) 1, Pd4, and Pd7). Given that collagen is the most abundant protein fibre in the dermis and that it provides strength and cushion, typically assisting the axon terminal, any changes in collagen morphology would likely reflect the existence of mechanical forces. Therefore, we focused on the changes in collagen fibres with regard to the morphological change in axon terminals at the apex of the dermal papillae (where RA-I is located). At Pd1, the axon terminals were mostly straight (Fig. 1A). At Pd4 and Pd7, the axon terminals exhibited a drastically altered shape with sinuousness similar to that in adult RA-I receptors (Fig. 1B,C). The development of RA-I receptors in this experiment was consistent with previous observations by light and electron microscopy^17^. On the other hand, the collagen fibres at the base of dermal papillae were dispersed at Pd1 (Fig. 1A). During the development process, the collagen fibres gradually became denser (Fig. 1B,C) regardless of the increase in finger size. The distance between neighbour collagen fibres was calculated (see ‘Method’ and Supplementary Fig. 4) and is presented in Fig. 1D. The mean distances between two neighbouring collagen fibres were 12.4 ± 4.7, 8.1 ± 3.4, and 6.2 ± 3.1 *μ*m for Pd1, Pd4, and Pd7, respectively (n = 200 for each individual). The calculation of the Mann-Whitney U test revealed significant differences between Pd1 and Pd4 as well as between Pd4 and Pd7 (*p* < 0.001). The difference between Pd4 and Pd7 was less significant than that between Pd1 and Pd4 (Fig. 1D). The change in dermal thickness was also calculated and is presented in Fig. 1E and Supplementary Fig. 5. The mean thickness was 114.6 ± 24.2, 98.6 ± 30, and 67.4 ± 34.1 *μ*m for Pd1, Pd4, and Pd7, respectively. The difference in means was significant between Pd1 and Pd4 (*p* < 0.01, Mann-Whitney U test), as well as between Pd4 and Pd7 (*p* < 0.001, Mann-Whitney U test). These results indicate that the collagen fibres were compressed during the development process.

### Hypothesis of morphological changes of axon terminal due to extrinsic mechanical force

**Figure 2:**
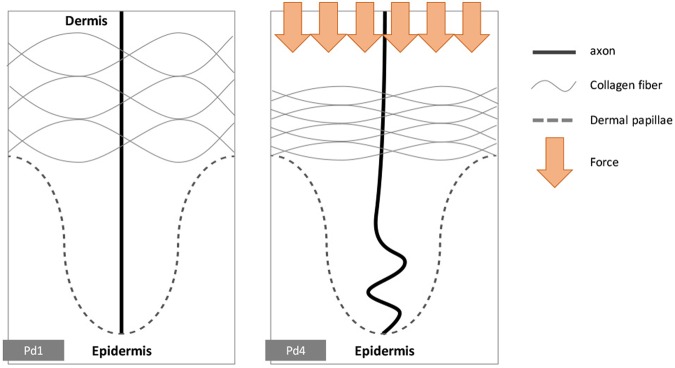
Hypothesis of the buckling behaviour of the first innervated axon at the first period of development (from Pd1 to Pd4). Note: The shape of the collagen fibres is illustrated to support the general concept only; their true profile *in vivo* is more complicated.

The decrease in the distance between two neighbouring collagen fibres and the dermal thickness suggest that they were compressed by an unknown extrinsic mechanical force during the periods from Pd1 to Pd4. This mechanical force may also affect the axon terminals of RA-I mechanoreceptors as they are supported by collagen fibres. The axon is a viscoelastic structure with estimated elastic modulus of 9.5 kPa^18,19^. The dermal environment surrounding axonal terminals in dermal papillae is also viscoelastic. Therefore, the axon terminal should theoretically buckle in the same manner as a thin elastic rod in an elastic medium when sustaining a compressive load from one end^20^. In short, the axon terminals of developing RA-I mechanoreceptors are hypothesized to buckle due to the extrinsic mechanical force (Fig. 2).

### *In vitro* buckling behaviour of the axon terminal of DRG neurons caused by extrinsic mechanical force

**Figure 3:**
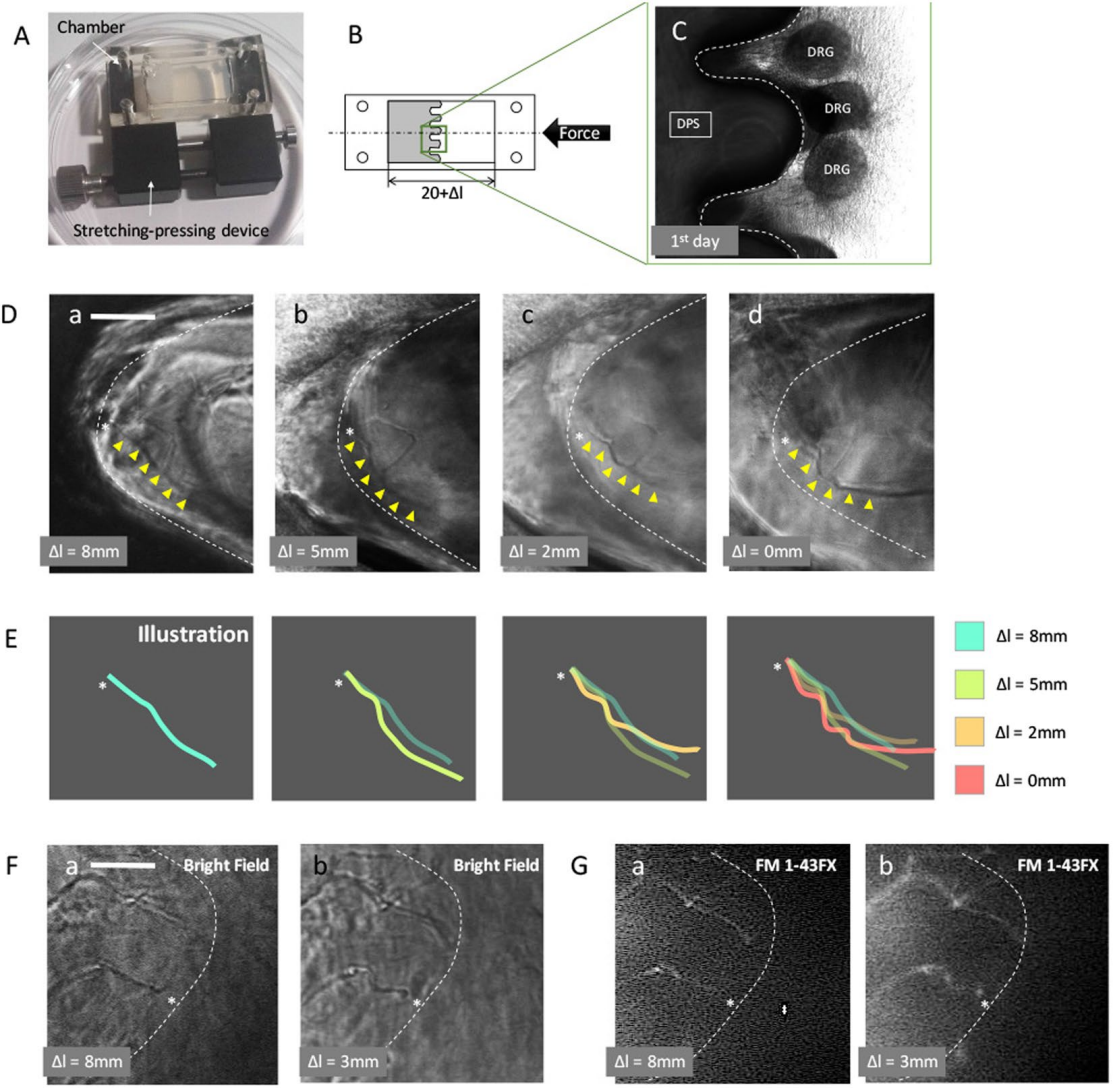
*In vitro* experiment with a stretchable chamber and mechanical pressing device. (**A**) Device setup. (**B**) Illustration of the compressing process. (**C**) Enlargement of the DRG positions at the initial stage; dashed white line indicates the boundary of the dermal papillary structure (DPS). (**D**) Sequential observations demonstrate the buckling of an axon at the apex of the DPS during compression. Bar: 30 *μ*m. (**E**) Illustrations of the buckling profile of the axon for better visualization. Each stage is encoded with a different colour. (**F**) Bright field observations of a compressed axon after treatment with FM1-43FX. Bar: 30 *μ*m. (**G**) The fluorescent profiles of the FM1-43FX treated axons in (**F**). The asterisks indicate the tips of axon.

To examine the effect of extrinsic mechanical force on the axon terminal, we used a stretching-pressing device as presented in Fig. 3A. DRGs were mounted on a skin model with dermal papillary structure (Fig. 3B,C). The chamber containing the skin model was initially stretched to a strain of 0.4. The calculation of strain is provided as *ε* = Δ*L*/*L*_0_, in which *L*_0_ is the original length of the chamber, and Δ*L* is the pre-stretched length. When the axon terminal reached the apexes of dermal papillae structure, the chamber was slowly released back to different rates of length as shown in Table 1. Using this method, the extrinsic force was provided to the axons unidirectionally. At each stage of release, care was taken to avoid the application of force in other axes onto the specimens. The observation demonstrates that the axon terminal began to buckle gradually as the strain decreased. At the initial stage, the innervated axon terminals were mostly straight and perpendicular to the silicone wall. The first curve of the buckling occurring at the axon terminal was observed at the 2nd stage, and the second and third curves were observed at the 4th and 5th stages, respectively (Fig. 3D). At the final stage, the sinuous profile of the axon terminal strongly resembled the morphology of RA-I receptors *in vivo*. Additionally, the measurements were similar to those of RA-I receptors in a mouse with the mean length of 40 *μ*m and a mean width of 10 *μ*m.

**Table 1:** Stages of observation and the subsequent length and strain.

Stages	Δ*L*	*ε*
*Initial*	8 mm	0.4
1	6 mm	0.3
2	5 mm	0.25
3	4 mm	0.2
4	2 mm	0.1
5	0 mm	0

Bright-field observation produces considerable noise, which may affect the final result. For better observation of axonal buckling behaviour and to further validate the results, we repeated the stretched-pressed experiment using culture medium mixed with FM1-43FX dye (N-(3-Triethylammoniumpropyl)-4-(4-(Dibutylamino) Styryl) Pyridinium Dibromide). The stained profiles were consistent with those observed by phase-difference microscopy (Fig. 3E,F). The buckling of the axon terminal is presented in both fluorescent observations (Fig. 3E) and light-field observations (Fig. 3F) at Δ*L* = 3 mm. To quantitatively evaluate the buckled shape of axon terminals, we calculated the sinuosity of axon terminals at two stages, namely, before and after compression. The procedure was described in detail in the ‘Method’ section and Supplementary Fig. 6. The buckled axon terminals were observed in 75% of the experiments (n = 12). Regardless of some unobservable axons after compression, further studies are required to more rigorously address the buckling behaviour of the axon terminal.

### Buckling of the axon terminal without dermal papillary structure

**Figure 4:**
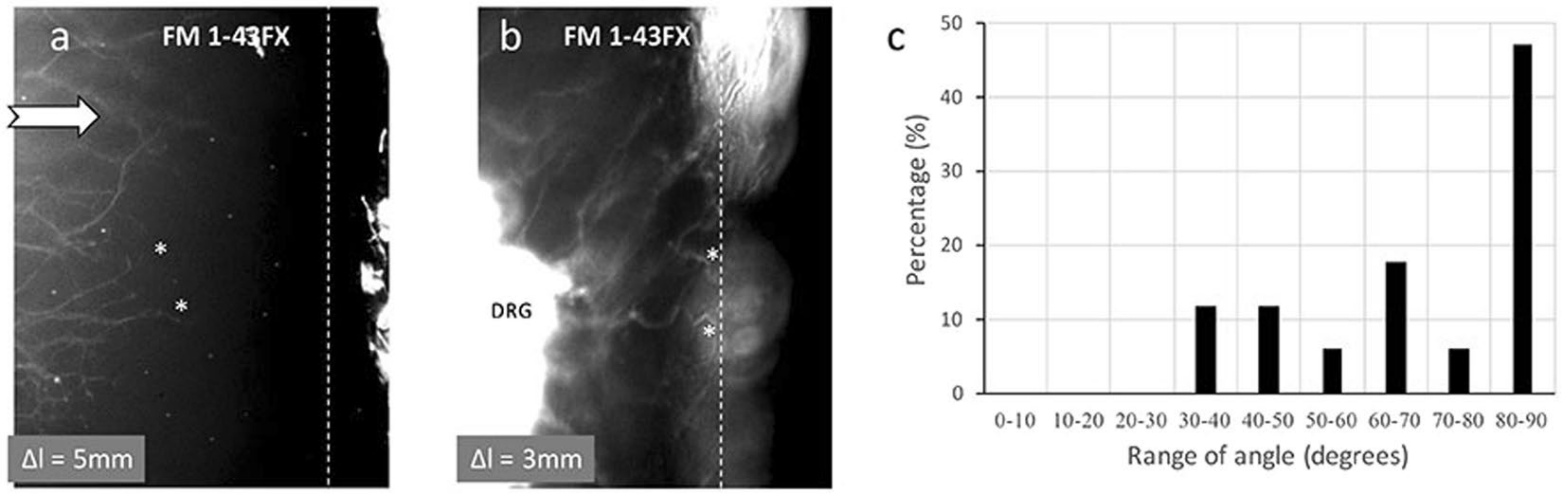
(**a**) Axons grow towards a flat silicone wall, staining with FM1-43FX dye at the initial state. (**b**) Buckling behaviour of axons after compression. The dashed white line indicates the wall boundary. The asterisks indicate the tips of considered axons. (**c**) Bar graph of percentage of buckling axons over a range of angles at which they grow towards the silicone wall.

Although the buckled axon terminals were observed in the apex of the dermal papillary structure, whether the dermal papillary structure itself was required for the deformation of the axon terminal is unclear. To answer this question, we performed an experiment where the DRGs faced a flat structure (Fig. 4a). An epidermis model without dermal papillary structure was fabricated and placed on the same initially stretched chamber using the same methodology employed in the previous experiment. After one day of culture, the axon terminals were observed to grow near the flat wall at various angles (Fig. 4a). A number of axon terminals buckled near the silicone wall when the strain was decreased to 0.15 (Fig. 4b), whereas other axon terminals appeared to slide in horizontal directions. By calculating the percentage of buckled axon terminals over a range of angles (Fig. 4c), we found that the axon terminals that were parallel to the force direction (perpendicular to the wall) exhibited the most buckling. The axon terminals that made an angle greater than 30 degrees to the force direction would slide rather than buckle. This result may help explain unanticipated axon terminals which were not buckled in the compression experiment with dermal papillary structure.

### Growth of the axon terminal may not cause buckling by itself

**Figure 5:**
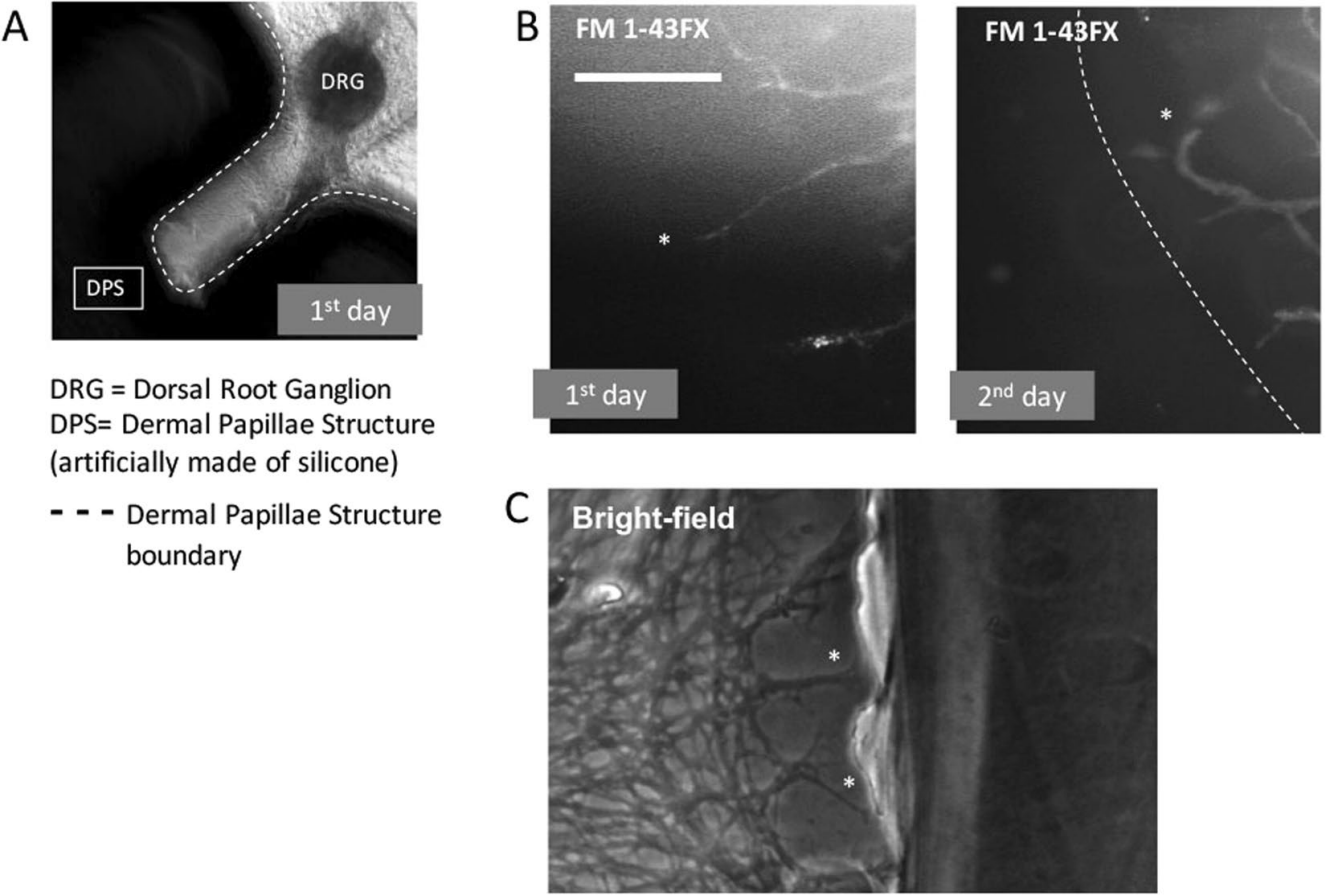
Growth behaviour of axons without extrinsic compressing force. The experiments were repeated 10 times. (**A**) Experimental setup with a ridged silicone wall. (**B**) FM1-43FX stained profiles of axons observed on the 1st and 2nd day of culture. Bar: 30 *μ*m. (**C**) Bright-field profiles of axons when in culture with flat wall. The asterisks indicate the tips of observed axons.

Finally, to examine whether the axon terminal could deform itself without extrinsic mechanical force after it reached the apex of dermal papillae structure, we monitored the neurons of the DRG in a long-term culture (10 days). The culture medium was carefully exchanged everyday to avoid the addition of any external force in the culture region given that previous studies^14^ have demonstrated the effect of fluid flow on the growth of axons. The DRGs were placed near the bases of dermal papillae structures (Fig. 5A). On the first day of the culture, the axons were straight when they reached the apexes of the dermal papillae structure (Fig. 5A). After one day, the axons were found to grow either along the silicone wall or backwards (Fig. 5B). Evidence of their growth along the silicone wall was observed in the experiment where the axons faced a flat wall (Fig. 5C). These results suggest that the intrinsic mechanical force may not be involved in the deformation of axons in this study.

### Quantitative analysis of the mechanical response of the surrounding environment during the *in vitro* compression experiment

Next, we quantitatively examined the effect of the extrinsic mechanical force on the buckling of axon terminals. However, direct measurement of the force occurring during the *in vitro* experiments, especially at the apexes of dermal papillae structure, is tedious. Therefore, we performed a simulation with an elastic finite element (FE) model, including the dermal papillae structure and collagen gel, with respect to their measurements. The model of the axon terminal was omitted for the sake of simplicity. In addition, we focused more on the stress that occurred in the extracellular environment than the stress on the inside of the axon terminal itself. The model was validated and then used to determine the magnitude and distribution of stress in the apexes of dermal papillae structures. This approach is illustrated in detail in Supplementary Fig. 1.

**Figure 6:**
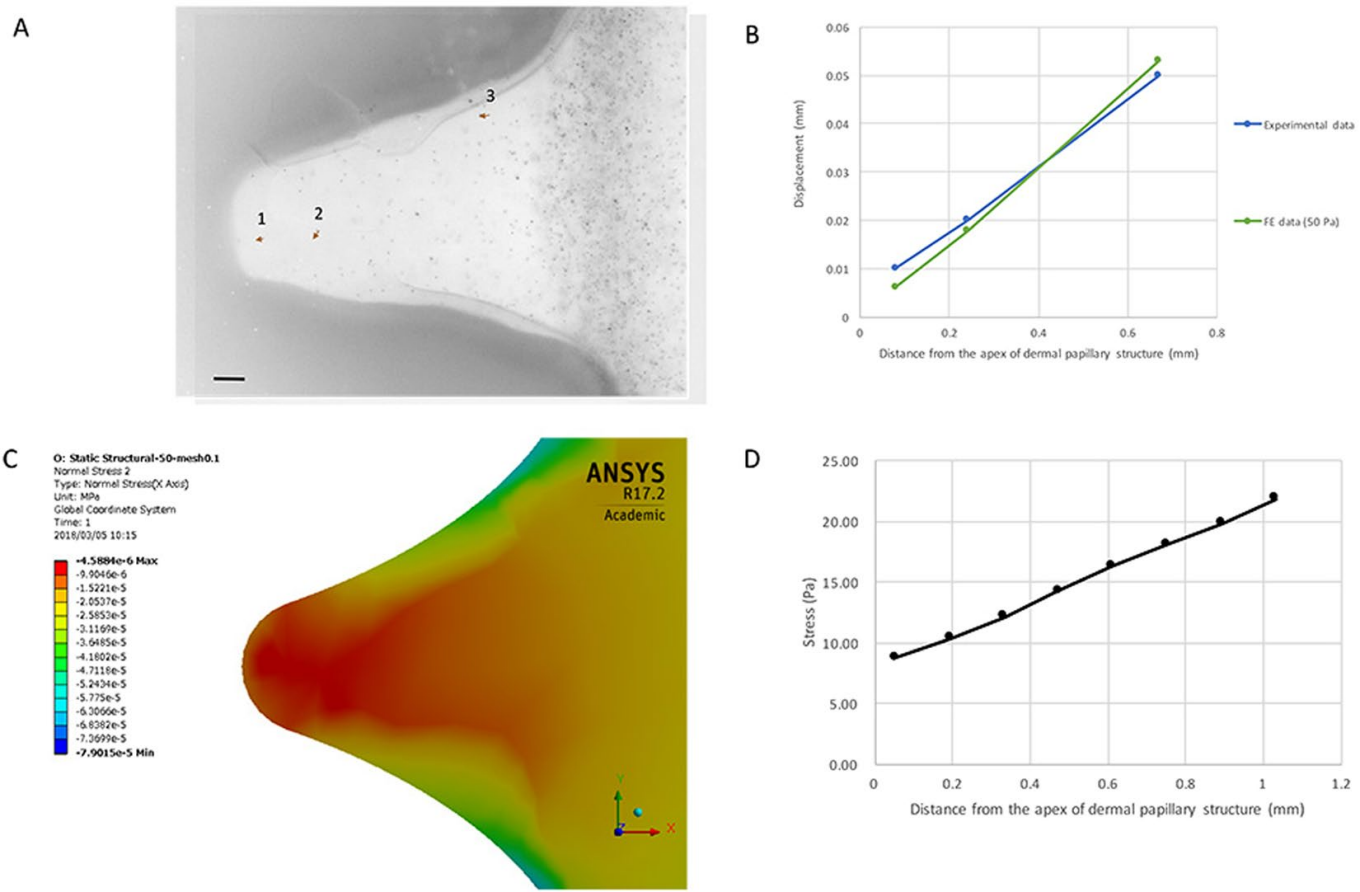
Simulation of the *in vitro* compression experiment. (**A**) Fluorescent bead displacement (red arrow) at one apex of the dermal papillary structure. The image was inverted for better visualization. Bar: 50 *μ*m. (**B**) The displacement of the selected position in experimental data (fluorescent beads) and the FE model. (**C**) The normal stress at the following apex of FE model. Colour bar indicates stress in MPa. The negative sign denotes that the direction of force is contrary to the direction of the axis. (**D**) The image depicts stress as a function of distance from the apex of the dermal papillary structure.

To validate the FE model, we measured the displacement of fluorescent beads poured into collagen gel at different stages during the *in vitro* compression process and compared these data with the calculated displacement of the following position in the model. Figure 6A presents the fluorescent beads and examples of calculated displacement at the apexes of dermal papillae structures after compression. The comparison of displacement demonstrated that the FE model was highly consistent with the *in vitro* experiment (Fig. 6B). As a result, the stress at the base of the dermal papillary structure (where the DRGs were located) was approximately 21.8 Pa. The minimum stress was 8.74 Pa at the apex of the dermal papillary structure. The stress increased proportionally to the distance from the apex of the dermal papillary structure (Fig. 6C,D).

To determine the minimal stress required for the buckling of the axon terminal, we then treated the axon as a bundle of microtubules (adding their flexural rigidity linearly). Theoretically, the critical force for the buckling of microtubules in an elastic medium is presented by the following equations^21,22^

1 $${F}_{cr}=8\times {\pi }^{2}\times \frac{\kappa }{{\lambda }^{2}}$$

2 $$\kappa =E\times \frac{\pi }{4}\times {r}^{4}$$

where *κ* denotes the bending rigidity, E denotes Young’s modulus of the axon, and r denotes the radius of the axon. We found that Eq. 1 describes the buckling of the axon terminal well, as shown in Fig. 3. Using the calculated stress and the measurement of the axon in Fig. 3 at ΔL = 8 mm (diameter of 4 *μ*m and length of 60 *μ*m) with the Young’s modulus of 144 Pa^23^, Eq. 1 provided a *λ* of 26.4 *μ*m, which is consistent with Fig. 3D. Therefore, the minimal critical stress for buckling of the axon terminal in this study was 0.9 Pa, which corresponds to *λ* = 2 × the length of the axon terminal (Euler buckling). Given that this value is significantly smaller than the calculated stress at the following position of the FE model, it additively validates the practicality of axonal buckling in this study. Interestingly, Eq. 1 also implies that a larger distance between the tip of the axon and the force application spot corresponds to a smaller critical force; thus, the axon terminal would buckle more easily.

## Discussion

Microtubules in living cells buckle on a short wavelength when they are pressed while being surrounded by cytoskeleton^21,22^. Given that microtubules are the main components contributing to the stiffness of an axon, the axon is expected to inherit their buckling behaviour. Various papers have promoted the idea where the axon, as a bundle of microtubules, may buckle under compression. Although supported by the results of mathematical simulations^24^, the buckling of the axon under compression has rarely been demonstrated experimentally^25,26^. In this study, we demonstrated that an *in vitro* extrinsic mechanical force could cause the deformation of axon terminals in the same manner as a developing RA-I receptor. The deformation process, in which the axon terminals are initially straight and then bend so that their lateral morphology becomes sinuous, is similar to those previously described for the RA-I developmental proces^17,27^. Of note, the dye FM1-43X used in this study inhibits the behavioural responses of sensory neurons to mechanical stimuli^28^. Therefore, the buckling of axon terminals presented in *in vitro* experiments was exclusively a physical phenomenon and occurred almost immediately after compression. This phenomenon is different from the physiological phenomenon where the mechanical stimuli enhances axon outgrowth by activating its mechanosensitive channels, like TRP channels^29^. With regard to such physiological phenomenon, the behavioural response of the axon terminal after being compressed for a period of time (for instance, a few days) will be investigated in our future work.

*In vivo*, a decrease in distance between two neighbouring collagen fibres in the dermis and a decrease in dermal thickness were observed, suggesting that the extrinsic mechanical forces (compression) are generated nearby the RA-I receptor in a period of time (from Pd1 to Pd7). The origin of the extrinsic mechanical force during the development of the RA-I receptor remains unknown. In a recent study, Seidenberg-Kajabova *et al*.^30^ observed volar pad prominence and regression (12^*th*^ and 13^*th*^ week in human foetus). Given that the bone maintained linear growth (see Supplementary Fig. 5), it is highly plausible that bone growth causes compression forces inside the skin during the regression of a volar pad. Alternatively, the continuing development of other deep layers in skin (for instance, subcutaneous tissues) that expands their volume may also provide stress in upper layers, such as the dermis.

Although our last *in vitro* experiment demonstrated that the axon may not become deformed by itself (intrinsic mechanical force) in the same manner as shown in the developmental process of the RA-I receptor from Pd1 to Pd4, it is important to note that the complexity of the inner activity of the axon should not be underestimated. Tamada *et al*.^31^ demonstrated that the rotation of growth cone filopodia can drive axonal turning. Additionally, to achieve the final spring-like shape (like matured RA-I receptors), rotational force is also necessary in addition to the compression force. The inner activity of the axon can be reasonably hypothesized to be the origin of this rotational force.

In future work, the bioengineering approach presented in this paper can be employed for reconstruction of desired RA-I mechanoreceptors *in vitro*. Our method uses a simple yet effective system, consisting of a mechanical device (3D printable) and a stretchable chamber. This approach is expected to help reveal a number of features and effects of the surrounding environment on neurons and RA-I receptors that have not been observed, for example, the underlying mechanism by which the RA-I mechanoreceptor continues to develop after Pd4.

Finally, a number of limitations in our approach at the present stage should be noted. Further investigations of the developmental aspect of RA-I mechanoreceptors requires improvements in our technique. First, the width between two apexes of the dermal papillary structure used in this study was large due to limitations of the laser cutter. A mould made by more advanced fabrication technology (for instance, photolabile hydrogel^7^ and 3D printer^32^) could achieve a smaller resolution, hence resulting in s a skin model with improved detail. A detailed skin model is required to address the effect of dermal papillary structure during the development of RA-I receptors. Second, future studies should introduce the co-culture of DRG and Schwann cells to obtain a better replica of RA-I mechanoreceptors. The protocol available in^33,34^ should be used to investigate the myelination. Third, to investigate the mechanotransduction of RA-I receptors, one should also consider neural recordings. Various techniques, such as electrode arrays, calcium imaging, patch clamp, and voltage sensitive dye proteins, are broadly used in neuroscience. A recent study^35^ demonstrated that DRG neurons are robust in culture, maintaining normal electrophysiological functions for a month. Non-contact methods, such as calcium imaging and voltage-sensitive dye proteins, could have an advantage compared with electrode array and patch clamp techniques given that these latter techniques typically require a stable culture environment. As mentioned above, the dye FM1-43X used in this study may inhibit the behavioural responses of neurons to mechanical stimuli, and future studies that involve the functional aspect of RA-I receptors should consider another dye to visualize the morphology of axon terminals.

## Conclusion

To conclude, we demonstrated that the axon terminal derived from dorsal root ganglion can buckle *in vitro* by an extrinsic mechanical force. This finding is important given that it helps explain a portion of the development process of RA-I receptors. *In vivo*, our results also suggested the presence of an extrinsic mechanical force nearby the developing RA-I receptor during this period of time through the reduction in both the distance between neighbouring collagen fibres and the dermal thickness. The source of this extrinsic mechanical force remains unknown. This bioengineering approach shows potential to be advantageously employed to study the developmental and functional aspect (mechanotransduction) of mechanoreceptors, the study of which is limited with traditional approaches.

## Methods

### Animal

Pregnant female mice (Mus musculus) were obtained from SLC (Hamamatsu, Japan; for ICR mice) or by mating in Nagoya University. Embryonic day (E) zero was defined as the day of vaginal plug identification. The Animal Care and Use Committee of Nagoya University approved all protocols for animal experiments. All experiments and methods were performed in accordance with relevant guidelines and regulations.

### Immunohistochemistry

Mice were fixed with periodate-lysine-paraformaldehyde (PLP) fixative. The distal pulp of mice was fixed in 4%PFA, immersed in 20% sucrose, embedded in Optimal Cutting Temperature (O.C.T) compound (Sakura Finetek USA, Inc.), and then frozen and sectioned perpendicularly to the longitudinal axis (18-*μ*m). Frozen sections were treated with the antibodies against PGP 9.5 (rabbit, UltraClone Ltd., dilution 1:400). After washes, sections were treated with Alexa Fluor 546-conjugated secondary antibody (A-11035, Life Technologies, 1:200) and subjected to two-photon microscopy (Olympus FV-1200MPE, Tokyo, Japan) using a 25X water-immersed objective lens (XLPNL25XWMP2, Olympus). Dermal collagen fibres were detected by autofluorescence obtained with emission at 450–550 nm^36^ by a wide-pass filter (FV10-MRV/R, Olympus).

### Measurement of the distance between collagen and the dermal thickness

The distance between two neighbouring collagen fibres was measured manually from the fluorescence microscopy images using the image analysis software, FIJI (Supplementary Fig. 4a). A number of straight lines connected the centre of the outer circle of the bone to distal points on the dermal papillae. Hence, each straight line crossed a number of collagen fibres (Supplementary Fig. 4b). The distance between two neighbouring collagen fibres was specified as the length of the segment connecting their cross-points with the same straight line. For each individual (Pd1, Pd4, and Pd7), the distance between two neighbouring collagen fibres was measured at 200 locations in total (5 cross-sections, 40 locations for each).

Similarly, the dermal thickness was specified with respect to the distance of two positions: the most proximal position to the bone and one point on the dermal papillae (Supplementary Fig. 5). The straight line connecting two points was perpendicular to the longitudinal axis of the collagen fibres. For each individual (Pd1, Pd4, and Pd7), the dermal thickness was measured at 36 positions in total (12 cross-sections, 3 positions for each). Given that RA-I receptors are typically concentrated at the pulp of the finger, the measured locations were limited to an area of 100 × 100 *μm*^2^ nearby the centre axis of the cross-section.

All statistical analyses were executed using Python programming language (Ver 3.5) and Python statistical libraries (stats, pandas and scipy packages). To reduce noise, all images were enhanced in contrast for 0.3% by FIJI software and converted into grey-scale beforehand.

### Preparation of the skin model

The model for the epidermis with papillae, which mimics the concave-convex dermal papillary structure, was created as follows. First, an acrylic mould was fabricated using a laser cutter (VLS2.3; Universal Laser System, USA) with a 10-mm × 20-mm bottom face and 2-mm depth (Supplementary Fig. 3). Given the limitations of the laser cut, the dermal papillary structure was 1000 *μ*m in height and 500 *μ*m in width. Silicone (RTV rubbers, KE45T, ShinEtsu Co., Ltd) was poured into the mould and incubated overnight at room temperature. Then, we carefully broke the mould and placed the silicone structure in 70% ethanol for two hours. Subsequently, UV light was used to sterilize the silicone structure for one hour. The epidermis model without papillae was created using the same methodology.

The dermis model was prepared as follows. Dorsal root ganglions (DRGs) of ICR mouse were used in this study. The DRGs were extracted from the mouse at E13 (Embryonic day 12) or E14 and preserved in medium (containing serum). A silicone chamber (STB-CH-04; Strex) was uniaxially stretched 40% using a manual-operated stretcher (STB-100; Strex). Then, the epidermis-like structure (model for epidermis) was placed at the fixed tip of the chamber. In the pre-stretched chamber, isolated dorsal root ganglions at E13 or E14 were mounted in collagen gel (AteloCell IAC-30; Koken) and cultured in DMEM/F12 containing horse serum (5%), foetal bovine serum (5%), N2 supplement (Gibco, 1:100), EGF (PeproTech, 10 ng/ml), bFGF (Invitrogen, 10 ng/ml) and NGF 2.5 S (N-240; Alomone Labs).

The entire chamber was covered by a 60-cm dish to protect the chamber from moisture and preserved in the incubator for 15 min. The culturing medium was added after the collagen gel had completely solidified. After one day, the chamber was relaxed to the original shape, thereby compressing the gel-embedded DRG neuronal axons (four stages, Table 1). Imaging was performed manually using an inverted fluorescence microscope (IX71; Olympus); microscopes were equipped with CCD cameras (Orca ER; Hamamatsu). To visualize cell morphology, DRGs were stained using the lipophilic fluorescent dye FM1-43FX (F35355; Molecular Probes).

### Identification of the buckled axon terminals

Hannezo *et al*.^37^ described the buckled form of the biological tube using four main categories: dilated, varicose, sinuous, and sausage-like. Given that RA-I receptors were mostly sinuous, the sinuosity was used to identify the buckled axon terminals in this study. The sinuosity of axon terminals was calculated using the following equation^25,38^

3 $$Sinuosity=\frac{b}{a}$$

where b denotes the actual length of the profile, and a denotes the shortest path length of the profile (straight line). The length of profiles was measured using the image analysis software. We adapted the threshold that was commonly used in geomorphology^38^. Therefore, a profile was considered straight if its sinuosity was less than a threshold of 1.06. In contrast, a profile with a sinuosity greater than a threshold of 1.06 was considered sinuous. Regarding RA-I receptor development, the axon terminals that shifted from straight to sinuous were specified as “buckled” in *in vitro* experiments.

### Visualization of the inner deformation of collagen gel under *in vitro* compression

Collagen gel with embedded fluorescent micro-beads (R0200; Thermo Scientific) was used to fabricate the dermis model. Then, the skin model was compressed under similar conditions (without DRGs), as in compression experiments of DRG axon terminals. Microscopic images before and after compression were obtained using an inverted fluorescence microscope (IX71; Olympus), and the displacement of microbeads was measured using FIJI.

### Simulation of induced force during compression

A finite element model of a stretchable chamber (containing collagen gel and silicone structure) was developed and validated based on the inner deformation of collagen gel (Supplementary Fig. 1). The analysis was performed using ANSYS software (version 17.2). The Young’s modulus of the silicone wall was calculated based on its official shore-A hardness (provided by supplier) using the following equation^39^

4 $$E=\frac{0.0981(56+7.62336S)}{0.137505(254-2.54S)}$$

where S denotes the shore-A hardness, and E denotes the Young’s modulus in MPa. The Young’s modulus of collagen gel was determined by trial and error. Seven Young’s moduli (20, 30, 40, 50, 100, 200, and 300 Pa) were tested. For each test, we measured the root mean square error (RMSE) between the displacement of fluorescent beads and the calculated displacement at their corresponding positions in the FE model. The RMSE is given by the following equation

5 $$RMSE=\sqrt{\frac{{\sum }_{i=1}^{n}\,{({\hat{d}}_{i}-{d}_{i})}^{2}}{n}}$$

where n denotes the number of samples, ${\hat{d}}_{i}$ denotes the displacement of *i*^*th*^ sample in the FE model, and *d*_*i*_ denotes the displacement of *i*^*th*^ sample obtained by observing fluorescent beads. In the final experiment, the Young’s modulus of collagen gel was set as 50 Pa, resulting in the smallest RMSE (Supplementary Fig. 2). Poisson’s ratio was 0.48 for both components, and both components were assumed to be incompressible. The FE model contains a total of 30,538 nodes and 8,704 elements. The analysis condition assumed the chamber to be slowly pressed at one end (from initial length of 28 mm to a length of 20 mm). In this case, the stress distribution is governed by the static translational equilibrium equation given by

6 $${\sigma }_{ij,j}=0$$

where *σ*_*ij*,*j*_ denotes the partial derivatives of the Cauchy stress tensor with respect to the Cartesian coordinates.

## Electronic supplementary material

Supplementary Information
