# Author Correction: Role of extrinsic mechanical force in the development of the RA-I tactile mechanoreceptor

**DOI:** 10.1038/s41598-018-30923-7

**Published:** 2018-09-07

**Authors:** Trung Quang Pham, Takumi Kawaue, Takayuki Hoshi, Yoshihiro Tanaka, Takaki Miyata, Akihito Sano

**Affiliations:** 1 Robotics Lab, Department of Electrical and Mechanical Engineering, Graduate School of Engineering, Nagoya Institute of Technology, Nagoya, 466-8555 Japan; 2 Pixie Dust Technologies, Inc, Tokyo, 101-0041 Japan; 3 Department of Anatomy and Cell Biology, Nagoya University, Nagoya, 466-8550 Japan

Correction to: *Scientific Reports* 10.1038/s41598-018-29390-x, published online 23 July 2018

The original version of this Article contained a typographical error in the spelling of the author Takaki Miyata, which was incorrectly given as Tataki Miyata. This has now been corrected in the PDF and HTML versions of the Article, and in the accompanying Supplementary Information file.

