# Supplementary material for: Role of extrinsic mechanical force in the development of the RA-I tactile mechanoreceptor
Source: Sci Rep. 2018 Jul 23;8:11085. doi: 10.1038/s41598-018-29390-x (PMC6056429; doi:10.1038/s41598-018-29390-x)
Supplement: Supplementary file 1 — Supplementary Information [file 41598_2018_29390_MOESM1_ESM.pdf]

<u>Supplementary Information</u>

Role of extrinsic mechanical force in the development of the RA-I tactile mechanoreceptor

Trung Quang Pham[1,*], Takumi Kawaue[3], Takayuki Hoshi[2], Yoshihiro Tanaka[1], Takaki Miyata[3], and Akihito Sano[1]

[1]Robotics Lab, Department of Electrical and Mechanical Engineering, Graduate School of Engineering, Nagoya Institute of Technology, Nagoya, 466-8555, Japan

[2]Pixie Dust Technologies, Inc., Tokyo, 101-0041, Japan

[3]Department of Anatomy and Cell Biology, Nagoya University, Nagoya, 466-8550, Japan

[*]To whom correspondence should be addressed

Email: q.pham.308@nitech.jp

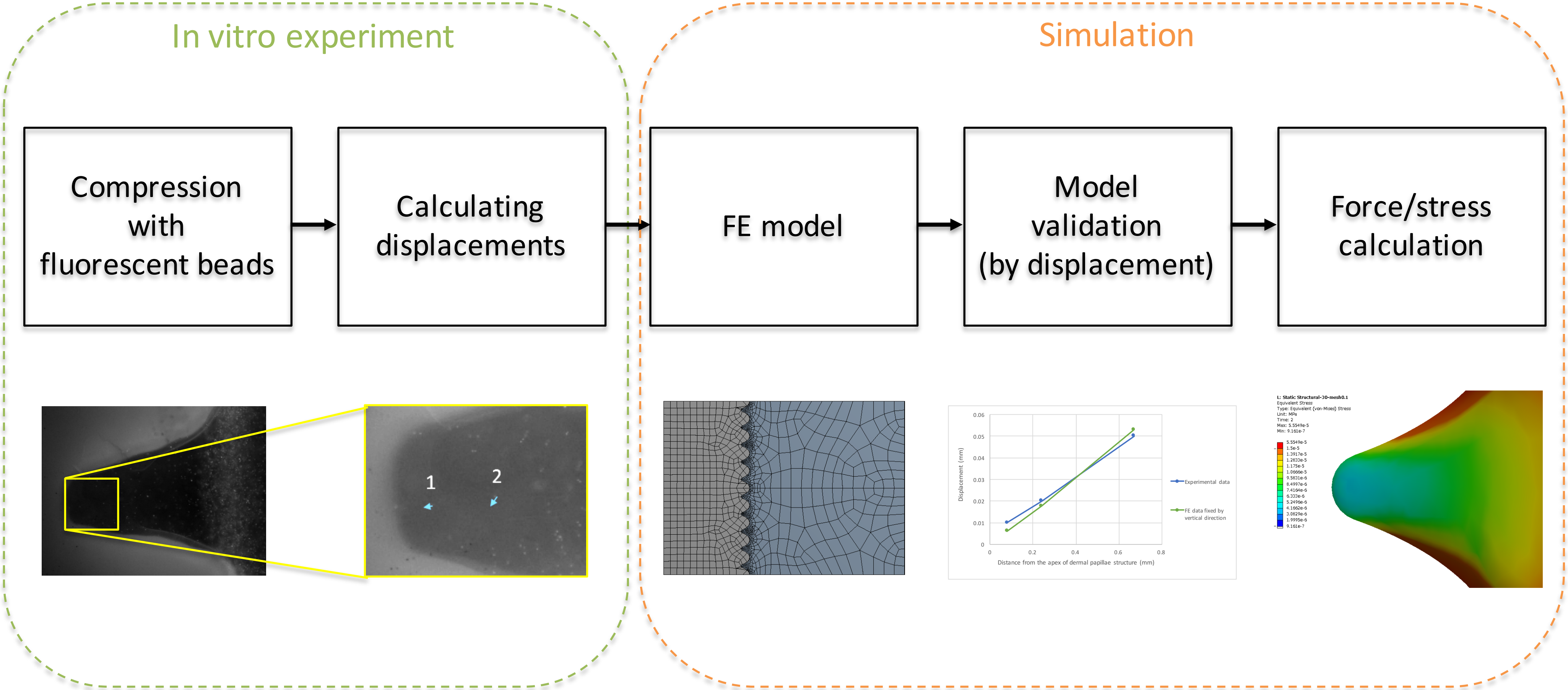

Supplementary Figure 1.
Determination process of inner deformation of collagen gel in skin model.

(a)

Young's modulus of collagen gel	RMSE
20	0.0036
30	0.0042
40	0.0032
50	0.0031
100	0.0042
200	0.0048
300	0.0047

(b)

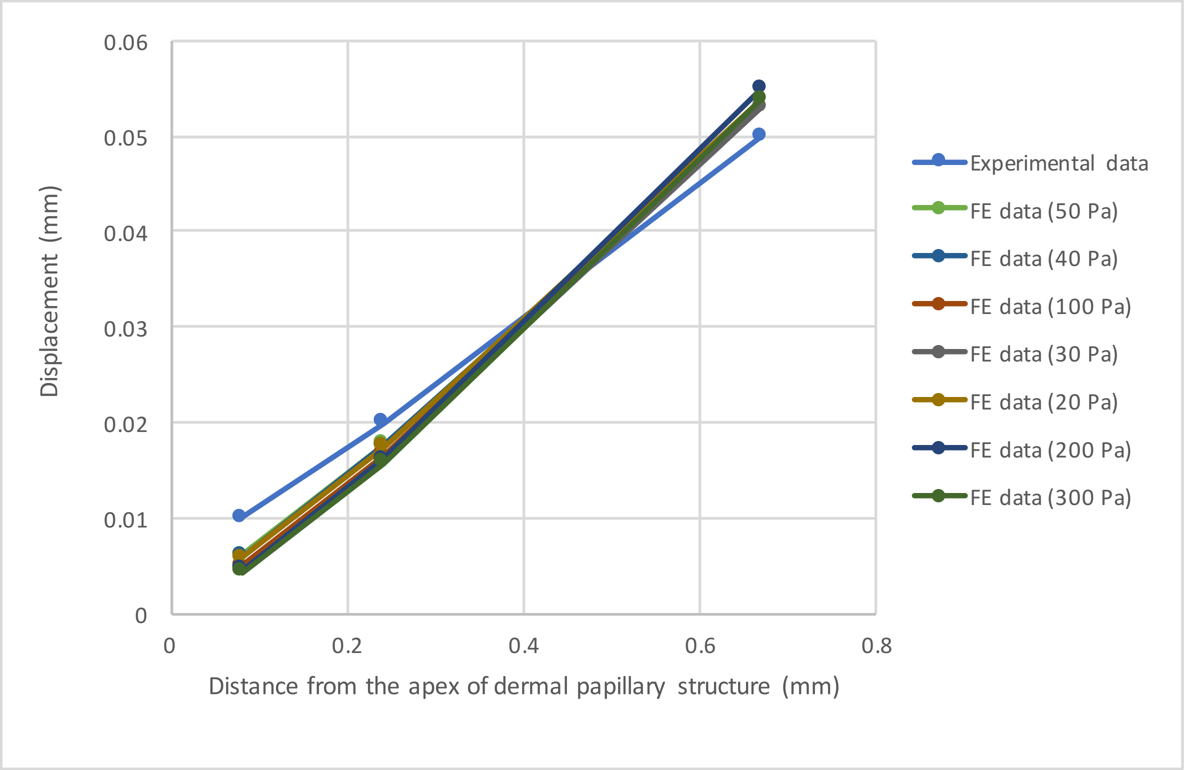

Supplementary Figure 2.

Determination of Young's modulus of collagen gel.

(a) Root Mean Square Error (RMSE) between displacement of fluorescent beads and corresponding positions in FE models. (b) Comparisons of displacement at corresponding positions in FE models

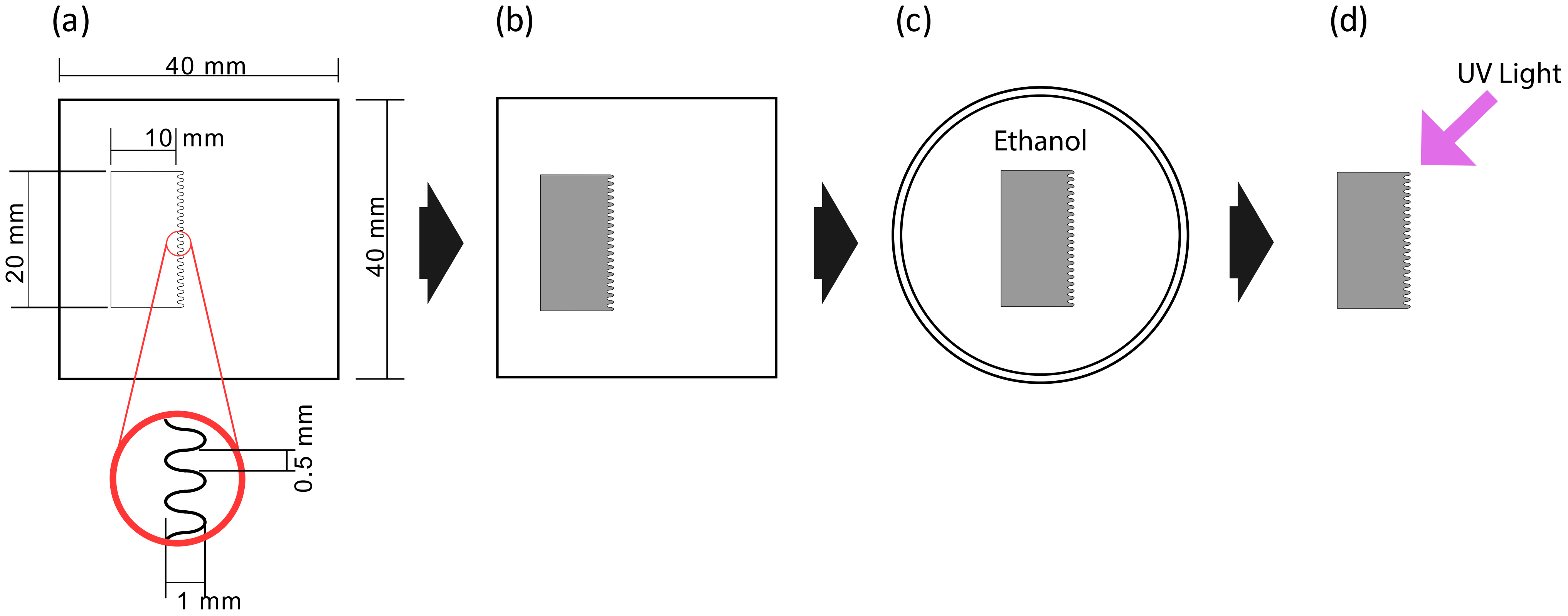

Supplementary Figure 3.

Fabrication of the model for epidermis with dermal papillary structure. (a) The mold prepared by a laser cutter. (b) Cast of silicone into the mold (model for epidermis). (c) Silicone (model for epidermis) was removed from the mold and immersed into 70% Ethanol for two hours. (d) Sterilization of silicone with UV light.

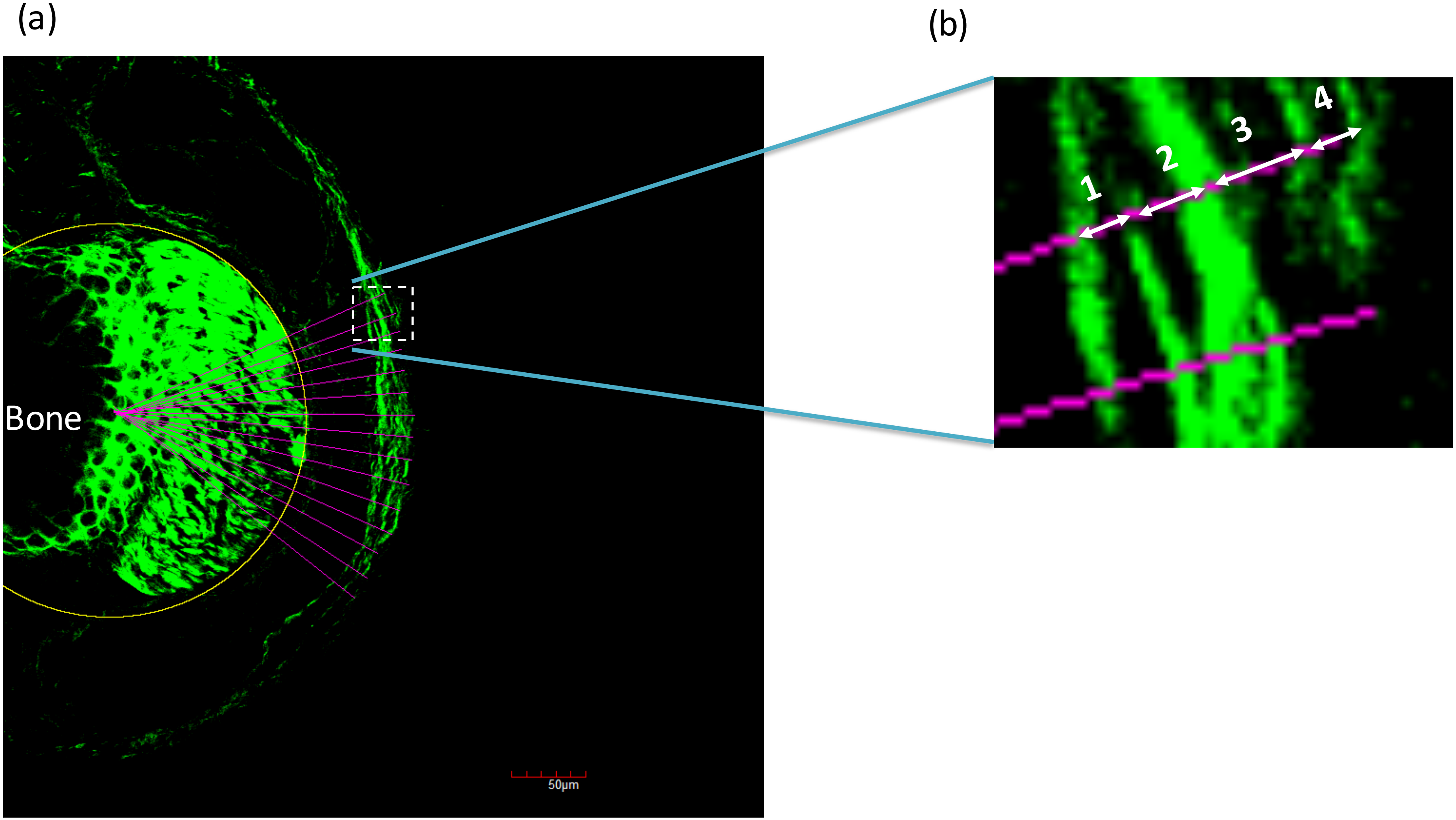

Supplementary Figure 4.
Measurement of the distance between two neighbouring collagen fibres. (a) An example of a measured cross-section (Pd7). The purple lines connect the center point of outer circle of the bone and the distal points on the dermal papillae. (b) Enlargement of the measured area. The white double-headed arrows depict the measured segments.

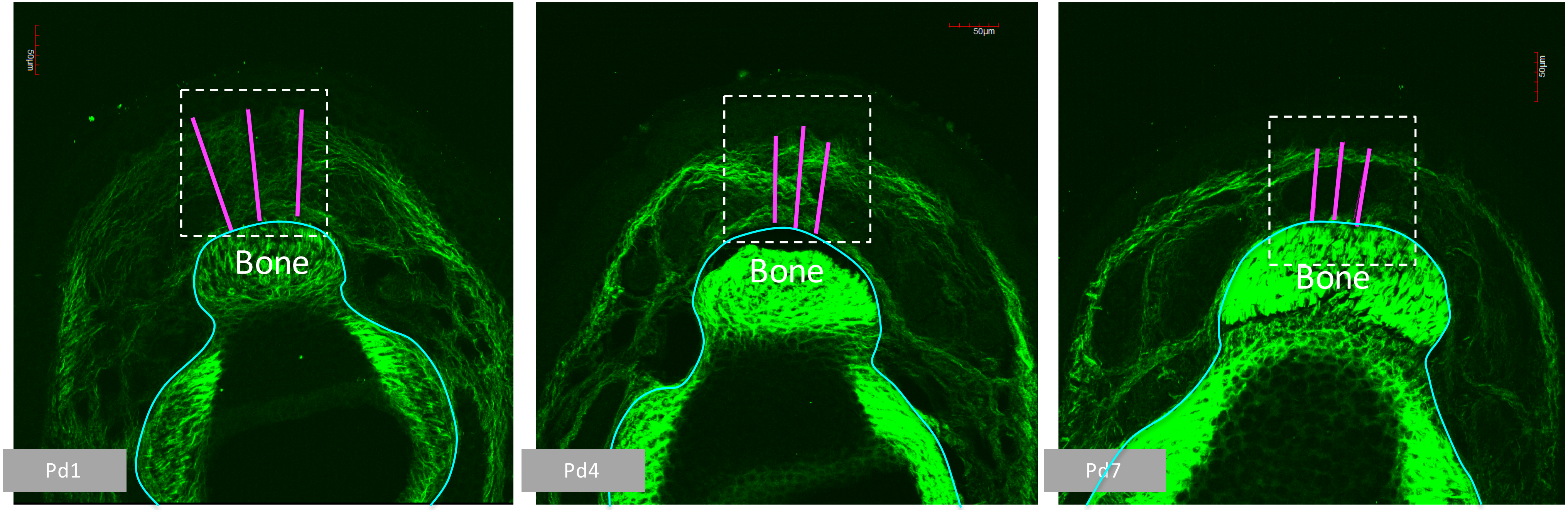

Supplementary Figure 5.

Measurement of the dermal thickness at Pd1, Pd4, Pd7 in the area of 100 × 100 µ*m*2 nearby the center axis of the cross-section (white dashed rectangles). The thin green profiles indicate the observing collagen fibres. The thin magenta lines indicate the measured distance from the phalanx's surface to the base of dermal papillae. The cyan lines depict the bone boundaries.

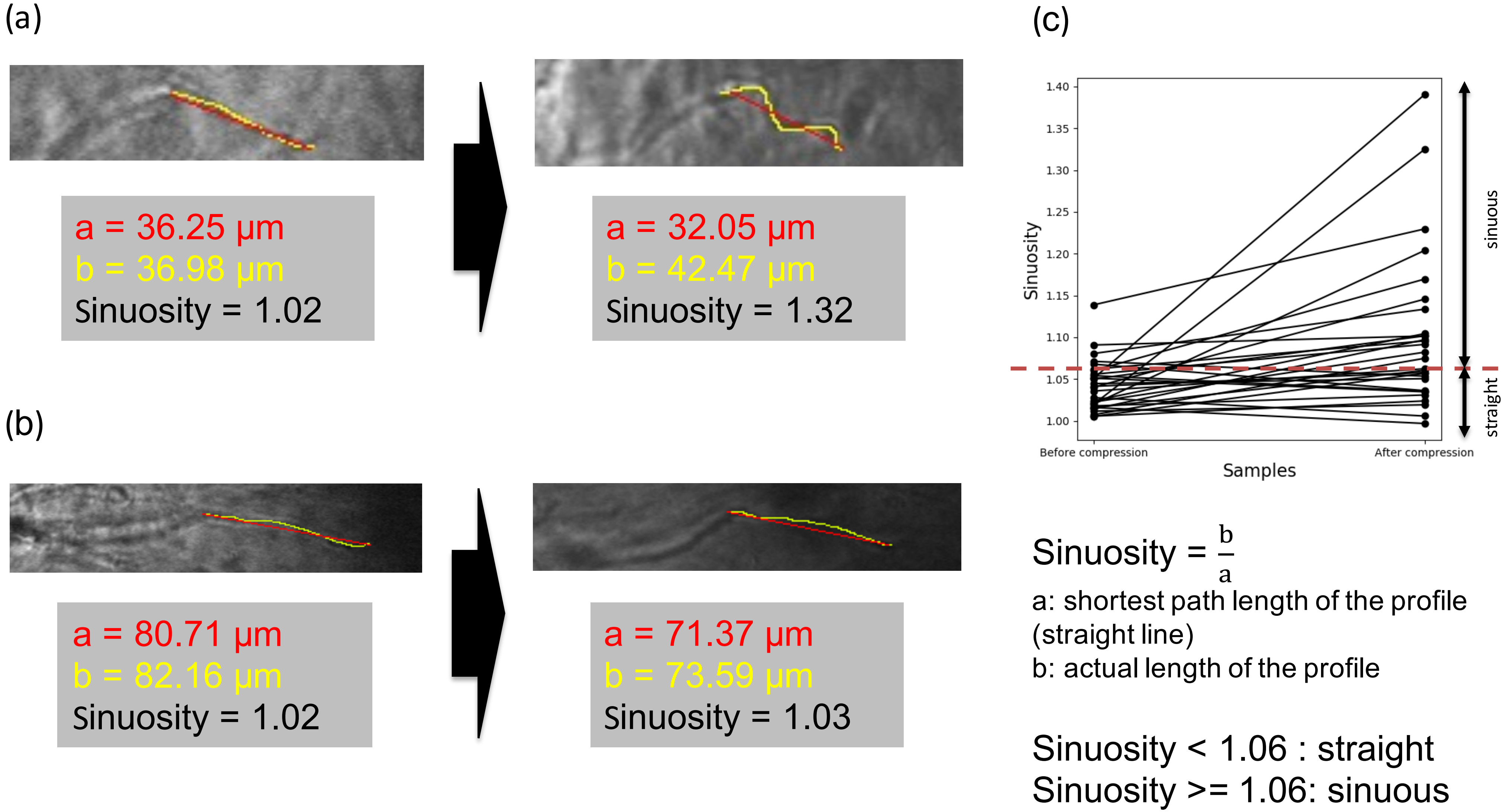

Supplementary Figure 6.
Calculation of sinuosity of the axon terminals. (a) An example of a buckled axon terminal. (b) An example of a non-buckled axon terminal. (c) Quantitative analysis of axon terminal's sinuosity before and after compression (29 axon terminals from 12 experiments). The red dashed line indicates the threshold (1.06) from which the axon terminals were considered straight or sinuous.
